# Dr. Dearborn

**Published:** 1884-06

**Authors:** 


### DR. DEARBORN.

The citizens of Chicago are familiar with the name of Dearborn. It is well known, in this locality at least, that the name was borne by General Henry Dearborn, and was in his honor given to the fort that occupies so large a space in the early history of this metropolis. But there are probably few medical men acquainted with the fact that the famous general was one whose military history was antedated by an honorable membership in their own profession.

Those who are interested in the precise facts to which we refer, should consult a scholarly paper on the subject of the Dearborns, prepared by Mr. Daniel Goodwin, Jr., of this city, lately read before the Chicago Historical Society, and now published in the proceedings of that body.

From this sketch, it appears that the Dearborn family in America began with one Godfrey Dearborn, born in Old Exeter, county of Devon, England, about the year 1600. He first came to the Massachusetts colony about 1639, and soon after this removal settled in Exeter, New Hampshire.

In the fifth generation of his family appears the name of Henry Dearborn, born at North Hampton in 1751, who grew up among the New England hills, a fine type of manly strength, agility, and beauty. "He was tall and straight, muscular and agile. He was noted as an unmatched wrestler, never having met his equal, and was an ardent sportsman. In all his journeys he carried his gun and rod and dog, and was an expert in cricket and ball until long past middle life. When not engaged in business or exercise, he was a constant reader, and was master of as good English as the War Department has produced. After going through one of the best schools in New Hampshire, he began and completed a full course of medical instruction under Dr. Hall Jackson, of Portsmouth, a distinguished surgeon in the army of the Revolution."

Dr. Dearborn, before the outbreak of the Revolutionary war, was established in the practice of his profession at Nottingham. Taking up the habits of the young men in his vicinity, he made himself familiar, in his leisure moments, with military evolutions and the science of making war. "The spirit of the mountains was stirred." The sentiments that in our own day have prompted many doctors to join the rank and file of a volunteer army, were then moving the men who took such active part in the foundation of the American republic.

On the 20th of April, 1775, Dr. Dearborn heard the news that war had been begun by the action of the British troops at Lexington.

He made no delay. With sixty of his townsmen, who only knew that Boston and its inhabitants were in peril, they marched one afternoon, with guns on their shoulders, from Hampton to Cambridge, a distance of fifty-five miles.

This was the beginning of the doctor's military career. He was soon commissioned as a captain, in the first regiment commanded by Col. John Stark. In ten days he had enlisted a company, and marched with them to Medford, carrying on his own person both gun and sword.

We have not space to detail further the exploits of the young doctor-officer, nor to follow the other steps in his interesting career. We might with interest go with him into action at Bunker Hill, or march with him in the campaign under Benedict Arnold at Quebec, during which for a month he was prostrated by illness, cared for alone by a French boy, in a hut.

What stirring scenes were those in which he played his active part! Very different indeed were his, from the battle-fields on which Count Rumford led his foreign dragoons against the enemy. Of the latter, we had occasion to speak in a late number of this journal, as another of the young New England physicians whom the stress of that Revolutionary crisis withdrew from the peaceful pursuits of their profession to the bloody fields of war. Rumford was every inch a Tory; Dearborn, a patriot of patriots. Rumford distinguished himself at Strasburg and Munich; Dearborn, at Ticonderoga, on the banks of the Hudson, at Valley Forge, Saratoga, Yorktown, and Monmouth; names inseparably associated with the patriotic sentiments of the people of this land. He was the personal friend of most of the great heroes of his day and cause, fighting successfully by the side of Stark, Arnold, St. Clair, Gates, Greene, Sullivan, and Washington himself. His career not only in the field, but in Congress, has occupied a proud position in the annals of this country's history.

The medical men of this America have little reason to be ashamed of the list of names they have contributed to the long roll of those who have distinguished themselves in other professions. Rumford, Warren, Dearborn—these were but the promise of the many who were to succeed them in a later war, where there were no fewer opportunities of showing to the world of what stern stuff they were made.

Chicago has named one of its finest avenues after the doctor-general, Henry Dearborn; and it is interesting to note that since it was first given, there has never been a proposal to change it.

